# Delayed Superficial Inferior Epigastric Artery Flap as a Robust Reconstructive Option for Penile Shaft Defects: A Case Report and Literature Review

**DOI:** 10.7759/cureus.94261

**Published:** 2025-10-10

**Authors:** Naveed Kamal, Obaidullah Obaid, Zarak Khan, Hamid Fazeel, Amber Azam

**Affiliations:** 1 Burns, Plastic and Reconstructive Surgery, Manchester University NHS Foundation Trust, Manchester, GBR; 2 Burns, Plastic and Reconstructive Surgery, Northwest General Hospital and Research Centre, Peshawar, PAK; 3 Plastic and Reconstructive Surgery, Salford Royal NHS Foundation Trust, Manchester, GBR

**Keywords:** penile injuries, penile reconstruction, penile trauma, reconstructive flap surgery, superficial inferior epigastric artery flap

## Abstract

Penile skin defects may result from burns, traumatic injuries, and necrotizing infections. Existing research recognizes the critical role of reconstructive options, which include skin grafting, local pedicled flaps, and free tissue transfer. However, this study aimed to report a unique case of a large full-thickness skin defect of the penile shaft and its reconstruction using the versatile delayed superficial inferior epigastric artery (SIEA) flap with good outcomes, which is a safe, easy, and robust option in difficult scenarios.

## Introduction

Penile trauma with skin loss is a reconstructive challenge, which often requires multiple complex surgeries to achieve both functional and aesthetic restoration [1]. While various options exist for managing genital skin loss, the goals of reconstruction remain the same [2]. The selection of an optimal technique depends on the extent of tissue loss, the patient's specific needs, and the surgeon's choice.

We describe utilizing the delayed superficial inferior epigastric artery (SIEA) flap approach. This flap is based on the SIEA, a branch of the common femoral artery that arises just below the inguinal ligament. It travels within the superficial fascia of the lower abdominal wall and provides a reliable axial blood supply to the overlying skin and subcutaneous tissue [3]. For larger penile defects, the delay technique provides reliable robust tissue coverage, making it a versatile solution for complex and large penile skin defects [4].

This technique offers a promising option for addressing these challenges and achieving satisfactory outcomes for patients.

## Case presentation

**Figure 1:**
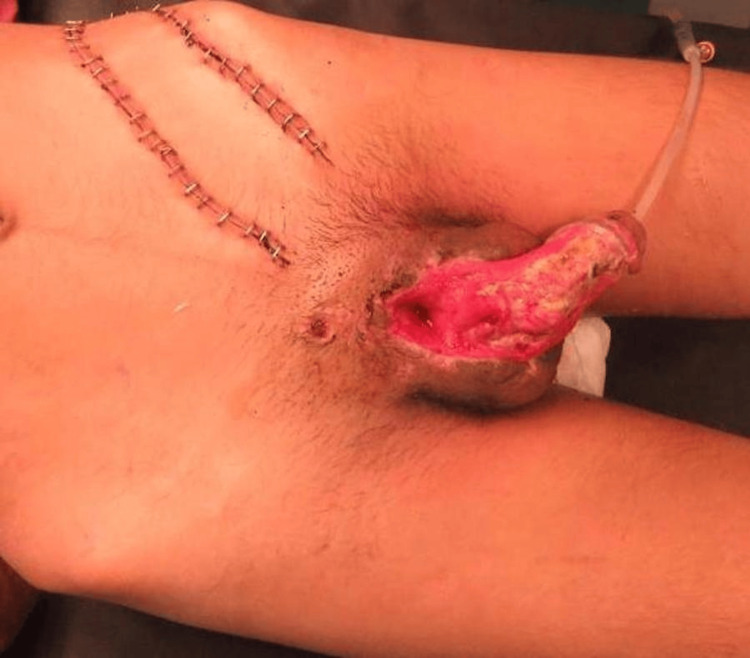
A large full-thickness skin defect over the penile shaft, extending from the root to the distal aspect of the shaft, sparing the glans, and a delayed SIEA flap SIEA: superficial inferior epigastric artery

A 21-year-old gentleman had an accidental firearm injury to his penis and right testis. The patient was stabilized and transferred to the plastic surgery department. Examination showed right testicular injury and a large full-thickness skin defect over the dorsal aspect of the penis, extending from the root to the distal aspect of the shaft, sparing the glans. The dorsal parts of the tunica albuginea of the corpora cavernosa were also exposed. Additionally, multiple rents in the penile urethra were also present (Figure 1). The aims for reconstruction were to restore a normal urinary stream and provide a supple coverage to the large defect with good functional and aesthetic integrity. 

The patient was admitted for surgery. The procedure was performed under standard general anaesthesia with the patient in the supine position. A penile block was then administered using 10 ml of 1% bupivacaine as a local anaesthetic. Wound debridement and thorough wash with normal saline were done. Methylene blue dye was injected into the penile urethra, which showed multiple rents in the penile urethra. The patient was catheterized with a 12-Fr silicone catheter. Subsequently, urethral rents were repaired using Vicryl 6-0 in an interrupted fashion. Right orchidectomy was performed in the same setting. 

The defect measured about 16×5 cm, which is more than 50% of the penile surface area. We anticipated a second debridement, so a staged procedure was planned. Furthermore, considering the full-thickness defect and exposed corpora cavernosa, flap coverage was considered rather than skin grafting. Accordingly, a left SIEA fasciocutaneous flap was designed and marked after the confirmation of the vascular pedicle using a handheld Doppler. The flap was delayed to recruit more vascularized soft tissue and skin to provide enough coverage to the defect. As the succeeding figures show, the incision extended superolaterally. Donor and recipient sites were dressed. 

**Figure 2:**
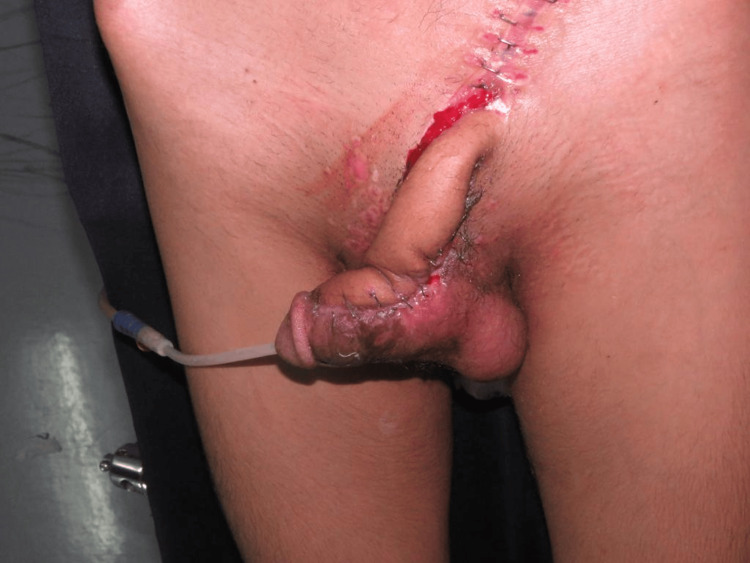
Healthy SIEA flap transfer and inset SIEA: superficial inferior epigastric artery

The second surgery was performed 48 hours after the first procedure. In this patient, a 48-hour surgical delay was considered sufficient due to a well-developed SIEA with a strong Doppler signal and the surgeon's clinical judgment and experience. As the early dilation of "choke" vessels usually begins within 48-72 hours, short delays have been shown to be effective in selected cases with favorable vascular anatomy. The wound bed was healthy, and flap vascularity was satisfactory. Flap transfer and inset were done. The donor site was closed primarily using staples (Figure 2).

**Figure 3:**
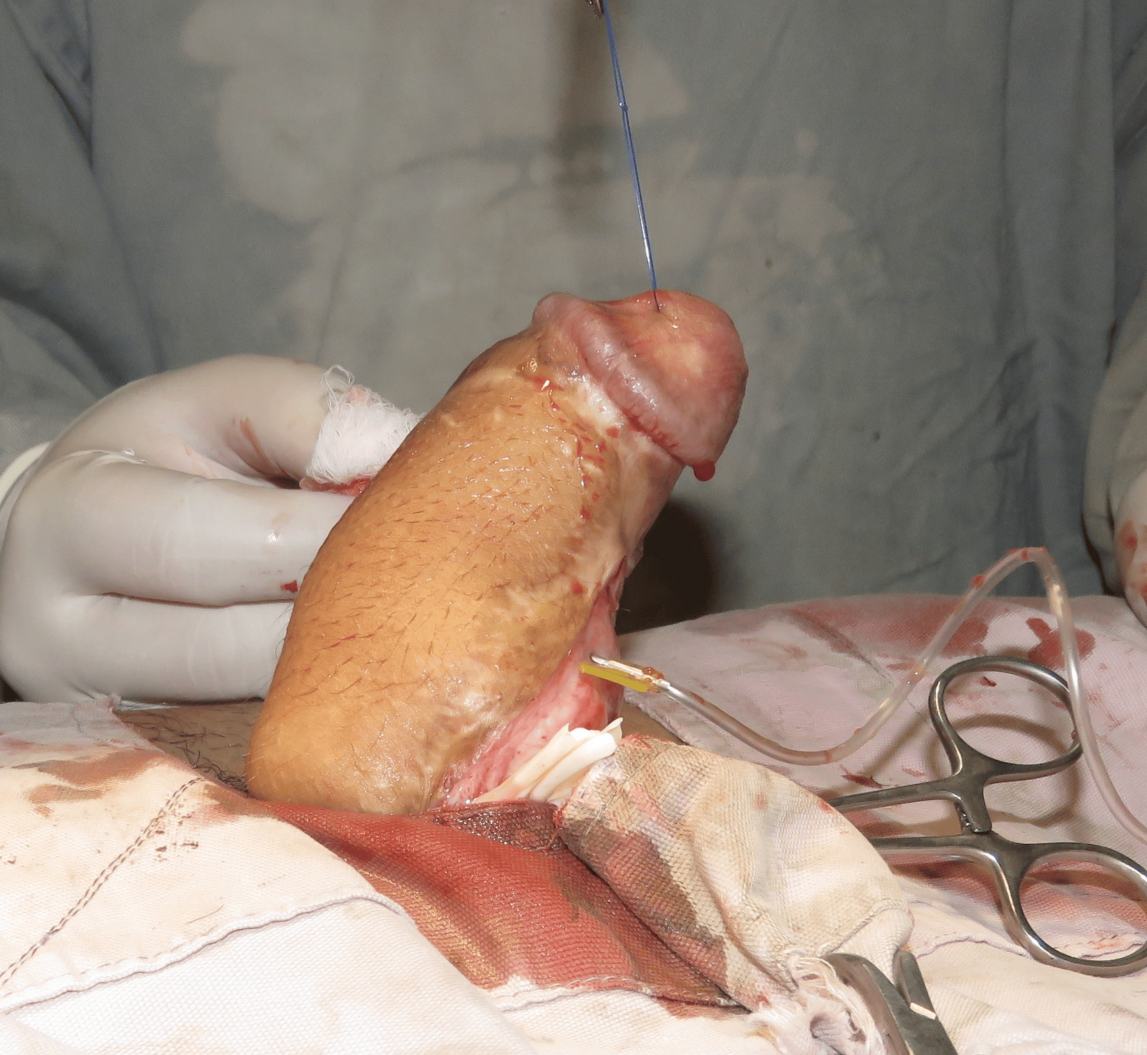
Artificial penile erection was performed by using a 24-gauge butterfly needle and 10 cc normal saline injection into the corpora cavernosa while applying a tourniquet at the base of the penis. Penile erection was observed with minimal chordee

Three weeks after the second procedure, artificial penile erection was elicited. While applying pressure at the base of the penis (Horton's test), penile erection with no significant curvature was observed with visual assessment. Flap division was done, and the donor site wound was closed primarily. The patient had an uneventful postoperative recovery (Figure 3).

**Figure 4:**
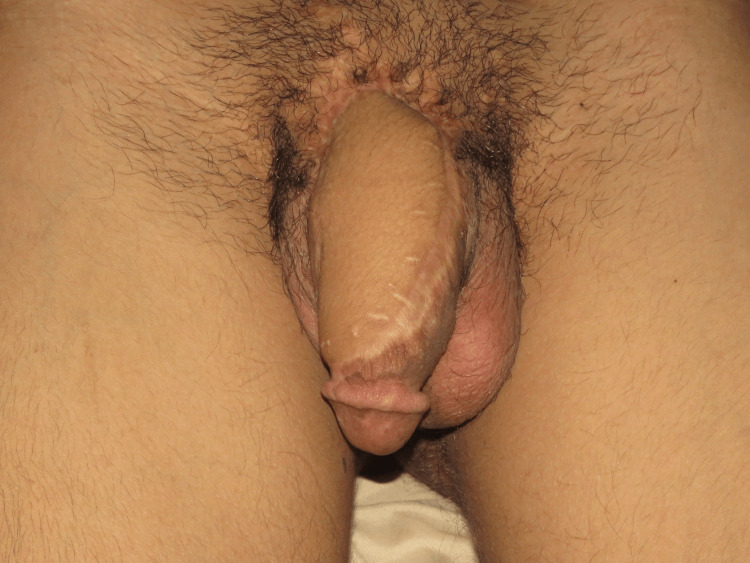
The patient at the two-year follow-up: anterior view. Good penile size, no secondary contractures, supple coverage, and comparable bulk

At the two-year follow-up visit, soft tissue bulk was comparable to the normal penile skin, and the patient had satisfactory voiding and normal erection (Figure 4). The maximal urinary flow rate was 20.3 ml/s with normal post-voidal residual urine.

## Discussion

The trend of reconstructive elevator approach in plastic surgery is gaining popularity in modern reconstructive surgery. However, the reconstructive ladder approach gives greater flexibility in handling difficult scenarios like this, especially in setups where resources are limited.

Alwaal et al.'s study [2] mentions that traumatic injuries and necrotizing infections are the leading causes of penile skin defects. Yazar et al. [3] demonstrated that the management of such patients is subject to patient condition and systemic and local factors such as the nature of the defect and goals of reconstruction. Zhao et al. [4] and Liguori et al. [5] combined debridement followed by negative pressure wound therapy, which has been proven to be an excellent option to reduce edema and enhance the wound healing process.

Maguiña et al. [6] and Demzik et al. [7] reported split-thickness or full-thickness skin grafts for reconstruction. In this case, these options were excluded because of the risk of developing a hematoma, which may jeopardize graft survival. Skin graft provides less pliable coverage with a high chance of urethra-cutaneous fistula formation, secondary contracture leading to penile shortening, and chordee development. Moreover, the full-thickness nature of the defect and the desire to provide supple coverage couldn't be achieved with grafting [1-6].

Other local flaps discussed by Zheng et al. [8], such as the superficial circumflex iliac artery flap, have been used by different surgeons. However, using a groin flap for reconstruction significantly limits the patient's postoperative mobility, whereas this limitation is not observed with the SIEA flap.** **Scrotal flaps result in hairy skin over the shaft. Here, it was ruled out because of the concomitant injury to the sac. The downside of the radial forearm flap is the loss of a major artery, an unattractive donor site scar, pain, and numbness. Lee et al. [9] successfully used the anterolateral thigh (ALT) flap in cases of severe necrotizing fasciitis; however, it is often too bulky, requiring further surgeries for debulking.

Furthermore, Musa and Ahmed [10] discussed staged penile reconstruction by groin flap, while Buitrago et al. [11] demonstrated reconstruction using the ALT flap. Other thin fasciocutaneous flap options for penile reconstruction include the scapular flap, radial forearm free flap (RFFF), ALT flap, and latissimus dorsi (LD) flap, which can be harvested as free flaps, typically considered when local or regional tissue options are unavailable or insufficient for penile reconstruction [11-13].

However, considering all the dynamics of this patient, such as multiple debridements and the size and depth of the defect, and avoiding the long-term complications of grafting, the best-suited option was a delayed SIEA flap.

Although the 16×5 cm SIEA flap is generally reliable along its axial vasculature, we performed a 48-hour delay in this case because the distal vascularity was uncertain. While a delay procedure can be beneficial for flaps harvested along a random or partially axial pattern to enhance perfusion via dilation of choke vessels [12], the delay served as a safe precautionary measure to enhance perfusion and ensure the reliable coverage of the penile defect while maintaining the advantages of thin, hairless skin and minimal donor site morbidity [13].

Postoperatively, excellent long-term results were noted. The patient has a normal urinary stream, satisfactory erection, and good aesthetic integrity in terms of skin color and bulk. However, the patient felt slightly decreased sensation over the dorsum. Regardless, no complications were reported at follow-up.

## Conclusions

The SIEA flap is a thin and pliable option. We have found it to be a robust, easy, and effective solution in difficult scenarios, especially in centers where microvascular free tissue transfer cannot be performed due to limited resources. Moreover, it can be a savior in case of a failing free flap. Nevertheless, a large number of cases are required to have a deeper insight into the use of this approach.
